# Probiotic Bacteria Survival and Shelf Life of High Fibre Plant Snack - Model Study

**DOI:** 10.1007/s11130-024-01196-5

**Published:** 2024-05-27

**Authors:** Marcin Kruk, Piotr Lalowski, Monika Hoffmann, Monika Trząskowska, Danuta Jaworska

**Affiliations:** 1 Institute of Human Nutrition Sciences, Warsaw University of Life Sciences (WULS–SGGW), Nowoursynowska 159c, 02-776 Warsaw, Poland; 2 Faculty of Human Nutrition, Warsaw University of Life Sciences (WULS–SGGW), Nowoursynowska 159c, 02-776 Warsaw, Poland

**Keywords:** Probiotic bacteria, No-dairy products, Antioxidants, bacteria survival, By-products

## Abstract

The study aimed to develop plant-based model snacks that are high in fibre, contain probiotic bacteria and are convenient for long-term storage. The research focused on selecting a suitable form of probiotic bacteria (active biomass, microencapsulated, freeze-dried), inoculation method (in the base mass or in the filling of a snack) and appropriate storage conditions (4°Cor 20 °C). The potential synbiotic properties were evaluated. The microencapsulated bacteria had the highest survival rate at 4 °C, while the freeze-dried bacteria showed better survival rates at 20 °C. Probiotics had a higher survival rate when enclosed inside snacks with a low water activity (a_w_ = 0.27) peanut butter filling than in snacks without filling (a_w_ = 0.53). Enclosing the probiotics in a low a_w_ filling ensures their survival at ambient temperature for 5 months at a count higher than 6 log CFU/g. The snacks exhibited high antioxidant capacity (average 300 mg ascorbic acid equivalent/100 g), polyphenol content (average 357 mg gallic acid equivalent/100 g) and high fibre content (average 10.2 g/100 g). The sensory analysis showed a high overall quality of the snacks (average 7.1/10 of the conventional units). Furthermore, after six months of storage, significant changes were observed in the antioxidant properties, polyphenol content and texture of the snacks, while their sensory quality remained unchanged. Moreover, a potential synbiotic effect was observed. The method used to assess bacterial growth indicated significantly higher values in the model snacks compared to a control sample. Therefore, this study has effectively addressed the gap in knowledge regarding the survival of probiotics in snacks of this nature.

**Supplementary Information:**

The online version contains supplementary material available at 10.1007/s11130-024-01196-5.

## Introduction

Modern life often exposes individuals to significant stress, leading to increased consumption of fast food and low-nutrient sweet snacks. A well-balanced diet is a critical determinant of health [1]. Fibre, an integral component of a balanced diet, significantly impacts the beneficial microbial fractions in the gut microbiota, which in turn affects the well-being of the host [2]. It is imperative to ensure the effective delivery of fibre and beneficial bacteria to the diet for the maintenance of homeostasis in the digestive system [1, 3]. Therefore, combining fibre and probiotic bacteria as a synbiotic is important to ensure appropriate modulation of the host gut microbiota and the development of probiotic bacteria supplied with food [2]. Fermented dairy products are a primary source of beneficial lactic acid bacteria [4]. However, the consumption of these products is limited because of dietary restrictions prohibiting dairy, short shelf life of products, or specific sensory characteristics that do not meet the sensory expectations of users [5]. Thus, there is a need to diversify the market for probiotic products that do not contain the above exclusions, provide dietary fibre, and have high consumer acceptance [6].

According to scientific literature, selecting the appropriate probiotic bacteria and fibre sources is critical when formulating synbiotic products. Factors in probiotic strain selection include storage temperature, form and method of bacterial inoculation, and viability of the probiotic strain in the final product [7]. In addition, introducing different sources of fibre into potentially synbiotic snacks is important. Mixing fibre fractions from various raw materials has been proven to increase the positive effect on the probiotic bacteria [8]. Therefore, to ensure proper differentiation of the prebiotic fibre fractions, authors decided to use various raw materials in this study such as dates, oat fibre, peanut butter and apple pomace. As an additional benefit, apple pomace is a waste material from the juice pressing industry and its waste level is still concerning because 25–30% is used as animal feed or fertilizer [9]. Incorporating apple pomace into synbiotic snack development could help reduce waste and have a positive environmental effect [10]. Moreover, snacks containing probiotics with a long shelf life have recently appeared on the market. However, there is a lack of scientific literature on the survival of these bacteria in such products and the methods for maintaining high bacterial counts in the finished product.

Consequently, the research aimed to create non-dairy snacks rich in fibre, fortified with probiotics, and enhanced with apple pomace. The investigation centred on three main aspects: (1) identifying the most suitable form of probiotic bacteria (whether biomass, freeze-dried, or microcapsules), (2) determining the optimal method for inoculation of the probiotics into the product, and (3) assessing the feasibility of storing the snacks under ambient conditions. The analysis encompassed evaluations of microbiological, sensory, physical, and chemical attributes. Furthermore, the study explored the potential synbiotic properties of the samples.

## Materials and Methods

This section is presented in the supplementary materials.

## Results and Discussion

In terms of basic chemical composition, two types of samples were analysed: samples with filling (C-F) and samples without filling (C-W). The contents of total fat, total fibre and its fraction are shown in Table 1. The total fat content was significantly higher in the C-F sample (*p* < 0.05). The fibre content was higher in the C-W sample due to the lower amount of fat in the composition (*p* < 0.05). Literature and reference data on fat and fibre content in dates, apples, peanut butter and apple pomace are similar to the results obtained [11, 12]. According to the manufacturer, the oat fibre used for the experiment contained 19 g /100 g of fibre, including 9 g /100 g of β-glucans.

**Table 1:** Total fat and fibre fraction content in tested samples (*n* = 3)

	C-W	C-F
	g/100 g	
Total fat	4.5 ^a^ ± 0.1	11.5 ^b^ ± 0.2
Insoluble fibre	8.7 ^a^ ± 0.0	8.4 ^b^ ± 0.1
Soluble fibre	2.0 ^a^ ± 0.0	1.4 ^b^ ± 0.2
Total fibre	10.6 ^a^ ± 0.1	9.8 ^b^ ± 0.1

Explanations: C-F- filled samples, C-W - samples without filling, letters a, and b show a statistical difference in the t-test (*p* < 0,05); descriptions of samples abbreviations are in the supplementary materials (Table S1); the value after the ± sign indicates the standard deviation, the statistical differences refer to compounds separately.

The total viable count of bacteria, moulds and yeasts was below 10 CFU/g in all tested samples. These results are presented in Supplementary Table S4. The micro-organisms were probably inactivated during the pasteurisation process of the raw materials.

**Fig. 1:**
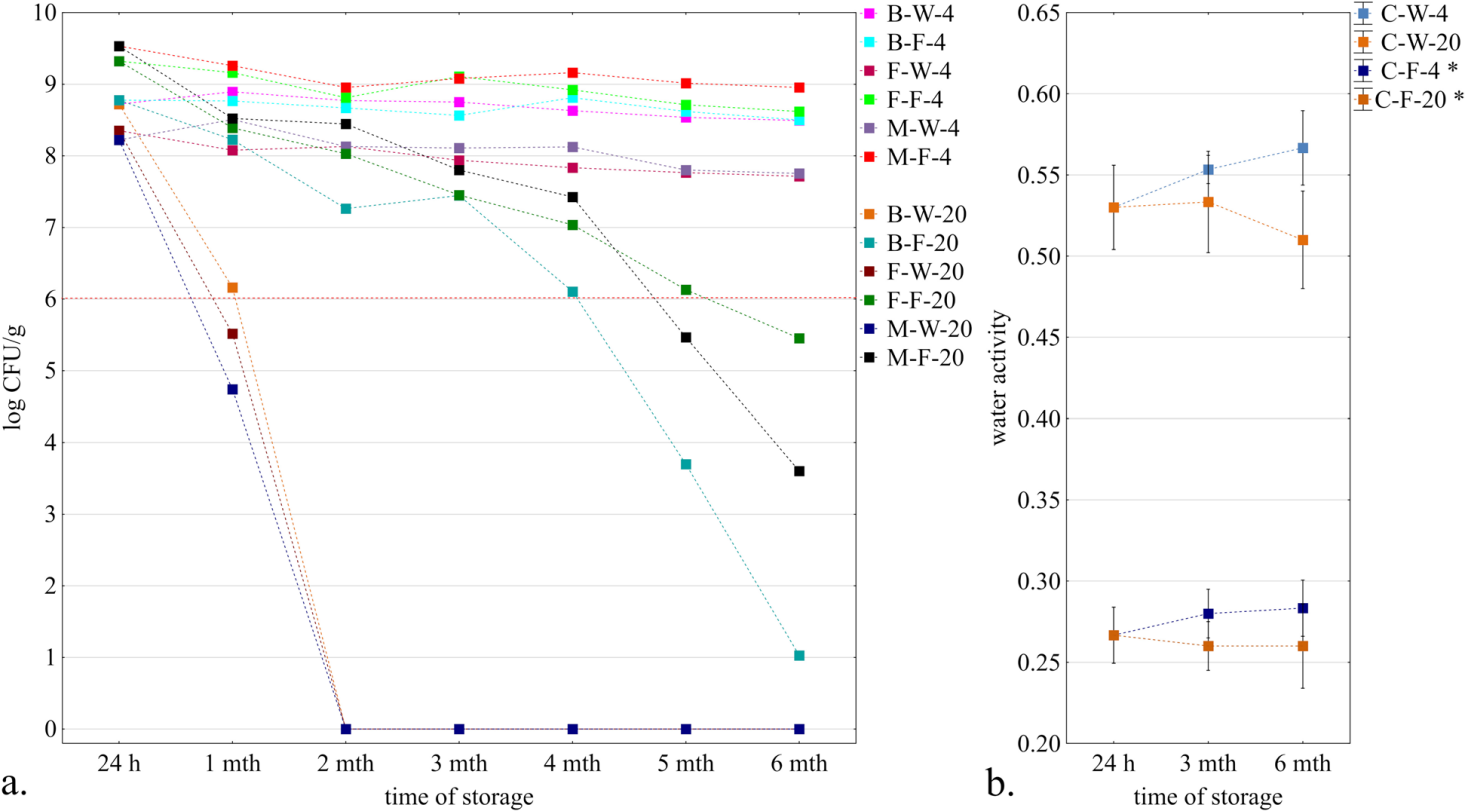
**a**, **b** Survival of bacteria in samples (**a**) and the water activity of the snacks and peanut butter filling (**b**) during storage; descriptions of samples abbreviations are in the supplementary materials (Table S1); * - water activity for peanut butter filling inside the sample, the water activity value for the date mass base did not differ significantly from the C-W sample; (*n* = 3); the red dashed line indicates 6 log CFU/g of the product; error bars indicate the standard deviation; h - hours; mth – month

The results of probiotic bacterial survival in model snacks are shown in Fig. 1a. During the 6 months of storage, the survival rate was similar in all variants stored under refrigerated conditions (4 °C). A significant decrease in probiotic survival was observed in samples stored at 20 °C (*p* < 0.05). After 2 months (20 °C), no probiotic bacteria were detected in the samples without peanut butter filling, regardless of the form in which they were added, i.e. biomass, freeze-dried or microcapsules. Probiotics survived better when peanut butter was used as a filling (*p* < 0.05). The initial water activity of the snack without filling was 0.53 ± 0.02 (Fig. 1b). Meanwhile, in the filled samples, the peanut butter enclosing the bacteria had a starting water activity of 0.27 ± 0.01. During storage, the water activity value of the samples without filling and filling from peanut butter did not change significantly regarding time and temperature (*p* > 0.05). Location bacteria in the peanut butter filling inside the snacks resulted in the viability above 6 log CFU/g after 4 months of storage (20 °C) in the samples M-F-20, M-W-20 and B-F-20. Nevertheless, after 6 months of storage, the number of probiotics decreased below the acceptable limit of 6 log CFU/g. Statistical analysis revealed that the time, storage temperature and type of sample were significant factors influencing the number of bacteria in the products (*p* < 0.05). The storage temperature had the greatest effect on reducing the survival rate of bacteria in the samples. Moreover, the sample type was an equally important factor influencing the survival of bacteria in products stored at ambient temperature (*p* < 0.05). After 6 months of storage in the samples, the bacteria were still alive, and the genetic sequencing exhibited its belonging to the species *L. rhamnosus* from all tested samples (supplementary materials - Table S3). This proves the lack of cross-contamination with other bacteria during storage. The literature indicates that storage temperature directly affects the rate of biochemical and molecular reactions in bacterial cell ageing. The higher the temperature, the more destructive the effect on cells [13]. For this reason, the reduction in bacteria population was greater in samples stored at 20 °C than at 4 °C. Furthermore, variations in bacterial survival were observed depending on the form of bacteria present in a peanut butter-filled snack stored at ambient temperature. It has been reported that spray-drying microencapsulation can damage bacterial cell membranes, ribosomes and regulatory proteins, thereby reducing their viability. In contrast, freeze-drying does not have such a detrimental effect on the cells, allowing them to remain viable [14]. Water activity also affects the viability of bacterial cells. Bacteria in the high range (1.0–0.9) show good metabolic activity, and nutrient transfer and excretion activities through a cytoplasmic membrane and cell wall function properly [15]. At lower water activities (0.85 to 0.35), inactivation and cessation of reproduction of microorganisms occurs [15]. This phenomenon was observed in the samples stored at 20 °C without peanut butter filling (a_w_ = 0.57), where all bacteria were inactivated after two months of storage. When water activity is less than 0.30, bacterial cells are dormant and metabolic activity is almost inhibited [16]. This property is used in food and industry to prolong the storage of bacteria, e.g. in freeze-dried form [16]. Peanut butter has low water activity due to its high fat and low moisture content [17]. A combination of low temperature and low water activity allows the long-term survival of microorganisms [17]. This mechanism was also observed in samples where probiotics were placed inside the peanut butter filling (a_w_ = 0.27). Therefore, the success of our study is the high survival of the probiotic in products with peanut butter filling stored, especially at room temperature.

**Fig. 2:**
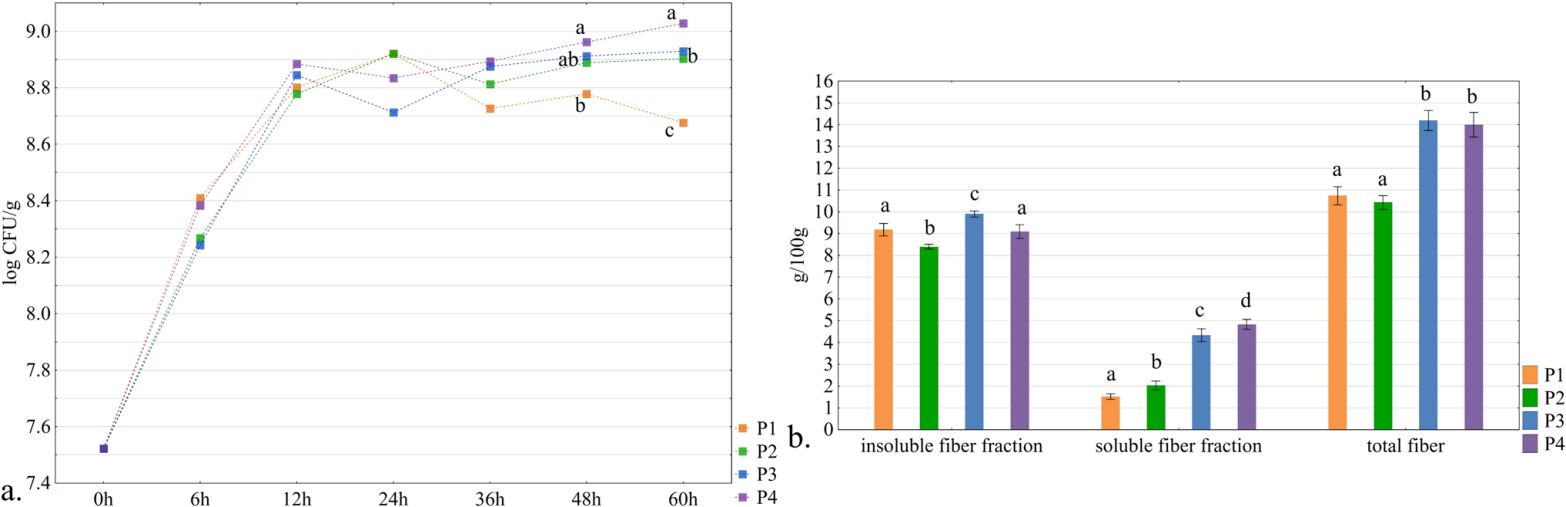
**a**, **b** Growth curves of *L. rhamnosus* ATCC 53103 in extracts from snack samples with various additional ingredients as a source of prebiotic substances (**a**) and the fibre content in tested samples divided into soluble and insoluble fractions (**b**); descriptions of samples abbreviations are in the supplementary materials (Table S2); (*n* = 3); letters a, b and c mean the statistical difference between the samples in the post-hoc Tukey’s test (*p* < 0,05), a. statistical differences relate to individual time points; b. the statistical differences refer to the fibre’s fractions separately; error bars indicate the standard deviation; h – hours

Regarding the findings on the potential effect of synbiotic snacks (Fig. 2). The consumption of synbiotics (probiotics + prebiotics) has a positive effect on homeostasis of human microbiome and it is essential to include them in the daily diet [2]. To demonstrate the synbiotic potential of a food, it must contain probiotics and prebiotics that stimulate their growth [2]. In this study, the analysis of dietary fibre content was divided into soluble and insoluble fractions (Fig. 2b). To observe the influence of these fractions on the change in the growth number of *L. rhamnosus* ATCC 53103, a 60-hour incubation was applied. This was because the fibre prebiotic fraction is catabolized by bacteria after the reduction of easily metabolised molecules such as monosaccharides or disaccharides [18]. The phenomenon of prolonged bacterial growth was observed in the samples with oat fibre (P2), apple pomace (P3) or both (P4), where the probiotic bacteria grew better than in the control sample (P1) (Fig. 2a). This result proves the presence of polysaccharides fermented after monosaccharides and other more easily catabolized energetic molecules. According to the literature, after metabolising simple energy sources probiotic bacteria initiate the enzymatic degradation of prebiotic substances [19]. Oat fibre and apple pomace are well-studied sources of prebiotic or potentially prebiotic fibre fractions. The prebiotic substance in oats is the β-glucan fraction, which directly stimulates the growth of probiotic bacteria [20]. Apple pomace contains potential prebiotic pectins with different molecular structures [21]. It was observed that mixing the prebiotic substances resulted in a higher stimulation of the growth of probiotic bacteria [8]. The combination of fibre fractions from two different sources prolonged the stationary (P2, P3) or logarithmic (P4) phase in the culture of probiotic bacteria.

**Fig. 3:**
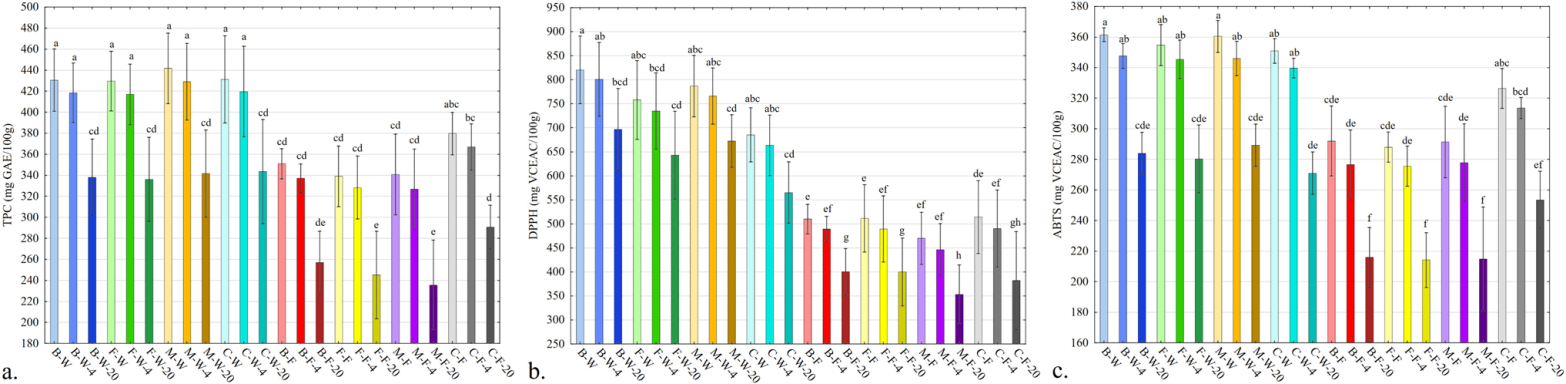
**a**, **b**, **c** Total polyphenol content (TPC) (**a**), and antioxidant activity expressed by scavenging radicals, DPPH (**b**), ABTS (**c**) of the tested snacks samples; descriptions of samples abbreviations are in the supplementary materials (Table S1); (*n* = 4); TPC results are expressed as gallic acid equivalent (GAE)**;** ABTS and DPPH results are expressed as ascorbic acid equivalent (VCEAC); error bars indicate the standard deviation; letters a, b, and c mean the statistical difference between the samples in the post-hoc Tukey’s test (*p* < 0.05)

The results of TPC and antioxidant activity are shown in Fig. 3. TPC and antioxidant activity were higher in the samples without peanut butter filling. Probiotics addition to the snacks did not significantly affect the TPC and antioxidant capacity (*p* > 0.05). However, the sample with the added biomass (B-W) had higher antioxidant properties in the DPPH evaluation than the other products (*p* < 0.05). The samples with a peanut butter filling and probiotics differed from those without filling and had lower TPC and antioxidant activity (*p* < 0.05). Storage time and temperature significantly decreased the polyphenol content and antioxidant capacity. The decrease of TPC and antioxidants in samples stored at 4 °C was nonsignificant (*p* > 0.05). The changes in the content of the compounds analysed were greater in samples stored at 20 °C (*p* < 0.05). Probiotic bacteria did not preserve polyphenols and other antioxidants in the samples (*p* > 0.05). The content of antioxidant compounds in peanut butter is lower than in dates and apple pomace. For this reason, the introduction of an extra dose of peanut butter in the filled samples decreased the content of polyphenols and antioxidant compounds. The high lipid and low polyphenol content effectively decreased the TPC and antioxidant capacity of the snacks with an additional portion of peanut butter filling [22, 23]. Concerning the scavenging of ABTS and DPPH radicals, different values (Fig. 3) were obtained in these methods. However, the difference between the methods was also found in other works and is caused by different reactions generated by ABTS and DPPH and differences in their sensitivity to other types of antioxidants [24]. Bacteria in freeze-dried or encapsulated form have extremely limited metabolism. As a result, the production of substances with antioxidant properties is virtually inhibited. This phenomenon is caused by depriving bacterial cells of access to water, which regulates enzyme activity, protein synthesis and cell reproduction [25]. Also, the probiotic carriers like maltodextrin, cellulose and sodium alginate during the freeze-drying and microencapsulation process are not substances with antioxidant properties, so they will not enhance these properties [26]. In the B-W sample (samples without filling), highly active metabolic biomass of probiotic bacteria was introduced into a stressful environment with a destructive water activity effect (aw = 0.53). Subsequently, the intracellular substances, including antioxidant molecules, were probably released from the bacterial cells into the environment. The water activity (aw = 0.53) indicated cellular stress and damage to the bacterial cell. It probably changes the permeability and integrity of the cell membrane due to the osmotic stress effect [27]. As a result, intracellular metabolites leaked out of the cell, causing the higher antioxidant capacity of the B-W sample. Probably, this phenomenon did not occur in the sample with peanut butter filling and bacterial biomass (B-F). It was due to the low water activity of peanut butter (aw = 0.27), which supports cell protection against osmotic and other environmental stresses. The mechanism of water activity for bacterial cells was discussed in detail in section 3.2. The changes in TPC and antioxidant capacity of the tested samples during 6 months of storage were typical for food products [28]. The higher the temperature, the greater the decrease in the value of polyphenols and other antioxidants in the treated materials [28]. It has been suggested in the literature that higher temperatures during storage accelerate the radical and oxidative degradation of compounds in food matrices, whereas low temperatures slow down the reactions [29]. This mechanism was observed in the present study.

**Fig. 4:**
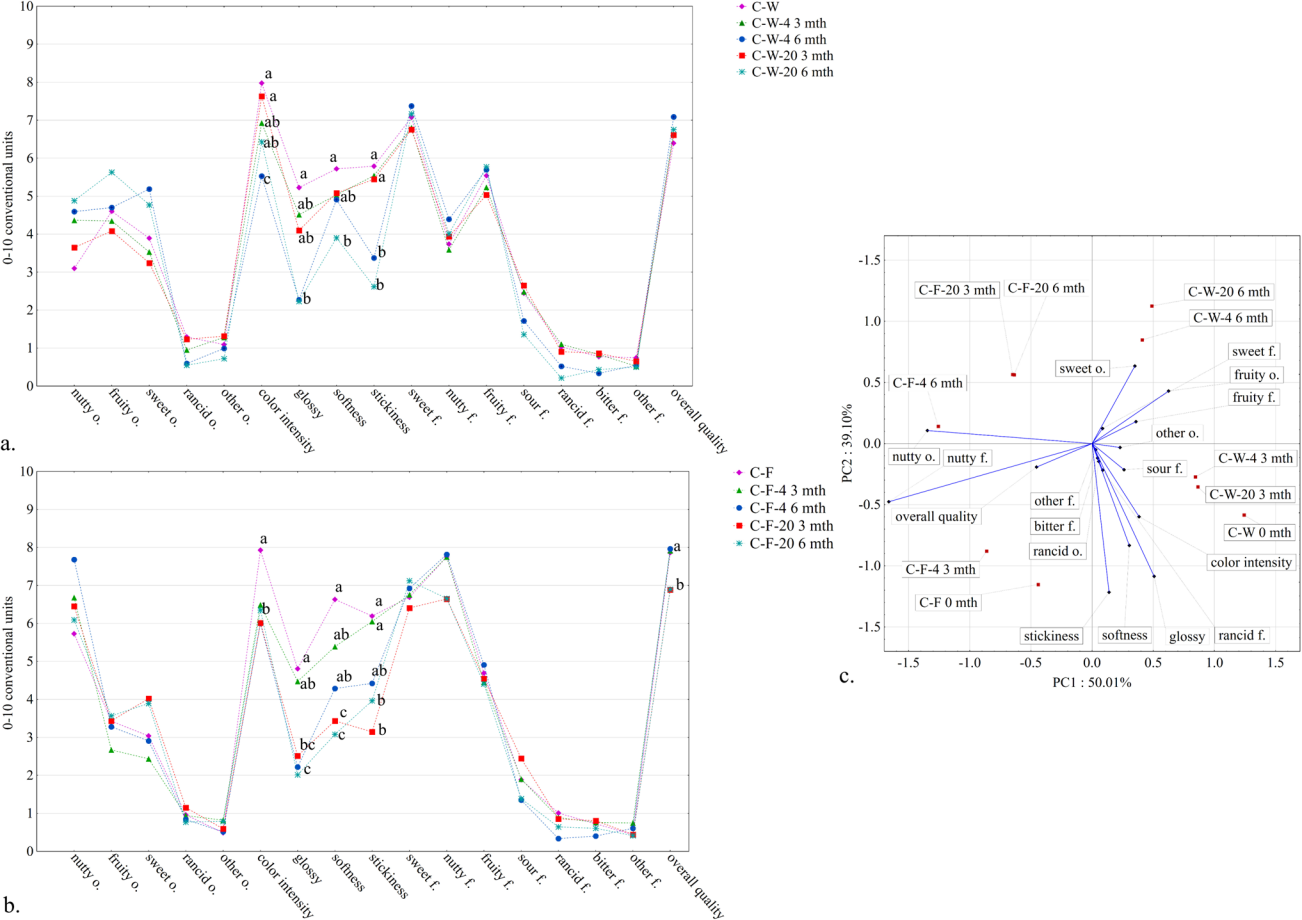
**a**, **b**, **c** Sensory profiles of the C-W (control sample without filling) (**a**) and C-F (control sample with the peanut butter filling) (**b**), after production (*n* = 16), after 3 and 6-months storage and, PCA of variables (sensory discriminants) and cases (tested samples) onto the plane of the principal components (PC1 and PC2) sensory discriminants and cases (tested samples) onto the plane of the principal components (PC1 and PC2) (**c**); full descriptions of samples abbreviations are provided in the supplemental materials (Table S1); letters a, b, and c mean the statistical difference between the samples in the post-hoc Tukey’s test (*p* < 0,05), statistical differences relate to individual sensory discriminants; o.: odour; f.: flavour; mth – month

Texture measurement results are shown in Table 2. Storage time significantly increased the cutting force and decreased the degree of deformation (*p* < 0.05). Storage temperature affected cutting force and deformation between C-W-20 and C-W-4 samples (*p* < 0.05). All the values obtained were higher in the samples without filling (C-W). The peanut butter filling caused a noticeable softening of the products. However, after 6 months all samples became harder than at the beginning. The low temperature limited water evaporation and allowed slower changes in hardness. In contrast, a temperature of around 20 °C favours water vaporisation and hardening of the samples [30]. The observed changes in texture are typical for the ageing of foods from this area [30, 31]. The changes in textural parameters agree with the sensory analysis results, where an increase in sample hardness was observed after storage (Table 3 and Fig. 4a, b).

**Table 2:** Texture measurements results of cutting force and deformation (*n* = 3)

Sample	Storage time	Cutting force [N]	Deformation [mm]
C-W	0 mth	13.5 ^a^ ± 0.9	24.6 ^a^ ± 0.4
C-W-20	6 mth	63.3 ^b^ ± 2.8	9.5 ^b^ ± 0.9
C-W-4		52.5 ^c^ ± 1.5	7.6 ^c^ ± 1.5
C-F	0 mth	8.5 ^d^ ± 0.1	10.2 ^b^ ± 0.4
C-F-20	6 mth	56.4 ^c^ ± 0.7	7.4 ^c^ ± 0.5
C-F-4		49.7 ^c^ ± 1.3	7.0 ^c^ ± 1.3

Explanations: descriptions of samples abbreviations are in the supplementary materials (Table S1); letters a, b, c, and d mean the statistical difference between the samples in the post-hoc Tukey’s test (*p* < 0.05); the value after the ± sign indicates the standard deviation; mth – month.

**Table 3:** Consumer acceptance of samples (*n* = 40)

	C-W	C-F
	9-point hedonic scale (1–9)	
Appearance	5.7 ^a^ ± 1.6	5.8 ^a^ ± 1.6
Consistency	6.6 ^a^ ± 1.5	7.1 ^a^ ± 1.2
Flavour	6.4 ^b^ ± 1.4	7.5 ^a^ ± 1.4
Overall liking	6.5 ^b^ ± 1.3	7.3 ^a^ ± 1.2

Explanations: C-W - samples without filling, C-F- filled samples, letters a, and b show a statistical difference in the t-test (*p* < 0.05); full descriptions of samples abbreviations are provided in the supplemental materials (Table S1); the value after the ± sign indicates the standard deviation, the statistical differences refer to sensory attributes separately.

Regarding the sensory study, the QDP analysis of the samples without peanut butter filling (C-W, Fig. 4a) shows that the tested material changed significantly in colour intensity, gloss, softness and stickiness during 6 months of storage at 4 °C. Storing at 20 °C changed the qualitative profile of the samples and reduced the sensory quality (*p* < 0.05). This was mainly due to the greater decrease in gloss and softness of the samples stored at 20 °C. The sensory quality of stored C-W samples was similar during 3 months, regardless of storage conditions. The same tendency was observed for the peanut-filled samples (C-F). Storage for 6 months at 4 °C resulted in fewer changes in the sensory profile of C-F samples (Fig. 4b) than C-W samples. The PCA analysis (Fig. 4c) showed that the overall quality of the snack was strongly and positively correlated with the nutty smell and taste. Softness was the most important attribute characterising freshness. In the PCA plot, the placement of filled snacks stored at 4 °C for 6 months indicates high sensory quality (close to the “overall quality” vector) (Fig. 4c). Furthermore, the closer position of C-F samples to the overall quality vector compared to C-W samples indicates their higher quality.

The consumer acceptance results (Table 3) show that the stuffed samples (C-F) were characterised by higher overall liking (*p* < 0.05) than the unstuffed samples (C-W). This resulted from a higher liking of the flavour (*p* < 0.05). Liking of appearance was at a similar level and not statistically different. There was no significant difference in consistency liking. As consumer demands and preferences are constantly changing, the sensory quality of food products plays a crucial role in measuring consumer response. A high sensory quality of food is one of the most important characteristics that determine the success of the product in the market and the consumer’s liking of it [32]. The mean value of more than 7 points (on a points scale from 1 to 9) obtained for the samples with fillings can predict the acceptance of the food products. In this study, the addition of peanut butter filling had a direct effect on the liking and sensory quality of the product. Sithole et al. [33] found that peanut butter positively affected the textural properties, odour, taste and overall quality of the food. Moreover, the peanut butter filling varied the texture of the snacks. It is defined that the complex texture of food products increases their overall sensory quality by intensifying the sensations resulting from consumption [34]. Adding peanut butter also increased the fat content of the snacks, which improved the flavour [35]. The higher consumer ratings of the product with a peanut butter filling and no major sensory changes during storage (QDP) may facilitate the easy introduction of the snack to the market. The filling improved sensory quality and allowed the product to be stored at room temperature for five months while still containing the recommended number of probiotic bacteria.

## Conclusion

The results demonstrated the possibility of developing a plant snack with high fibre content and probiotic bacteria. The findings suggest that the freeze-dried form of probiotic bacteria is optimal. However, their viability was highest when introduced into a snack with low water activity (a_w_ = 0.27). Storage conditions were identified as critical, with bacteria surviving at room temperature for up to five months at levels exceeding 6 log CFU/g. This required the probiotics to be in a low water activity environment and freeze-dried form; otherwise, survival rates diminished. Notably, snacks exhibited a high antioxidant content, dietary fibre, and superior sensory quality, with robust sensory including textural stability. Moreover, a positive probiotic growth response was observed in a model assessing growth stimulant presence, suggesting potential synbiotic properties.

Whilst yielding valuable insights, the study faces several limitations requiring future attention. Future investigations should delve into modulating intestinal microbiota through human studies and advanced in vitro models. Moreover, research is needed to assess levels of peroxides and other chemical compounds formed during storage, alongside exploring alternative probiotic strains with potentially higher survival rates. However, this study has effectively addressed the gap in knowledge regarding the survival of probiotics in snacks of this nature. Its findings hold potential for straightforward implementation within food processing.

## Supplementary information

ESM 1 (DOCX 88 kb)

## Data Availability

Data will be made available upon request to the corresponding author.

## Abbreviations

ABTS: diammonium 2,2′-azinobis[3-ethyl-2,3-dihydrobenzothiazole-6-sulphonate] radical
CFU: colony formation units
DPPH: 2,2-diphenyl-1-picrylhydrazyl radical
F-C: Folin–Ciocalteu
GAE: gallic acid equivalent
PBS: phosphate-buffered saline
PCA: principal components analysis
QDP: Quantitative Descriptive Profiling
TPC: total polyphenol content
VCEAC: ascorbic acid equivalent
