# Supplementary material for: Probiotic Bacteria Survival and Shelf Life of High Fibre Plant Snack - Model Study
Source: Plant Foods Hum Nutr. 2024 May 27;79(3):586–93. doi: 10.1007/s11130-024-01196-5 (PMC11410916; doi:10.1007/s11130-024-01196-5)
Supplement: Supplementary file 1 — (DOCX 88 kb) [file 11130_2024_1196_MOESM1_ESM.docx]

Supplementary materials for:

**Probiotic bacteria survival and shelf life of high fibre plant snack - model study**

Table of contents

[1. Abbreviations 2](#_Toc163921425)

[2. Materials and methods 2](#_Toc163921426)

[3. Results (DNA sequencing, moulds, yeast and total viable count of bacteria) 13](#_Toc163921427)

# **Abbreviations**

ABTS - diammonium 2,2'-azinobis[3-ethyl-2,3-dihydrobenzothiazole-6-sulphonate] radical; CFU - colony formation units; DPPH - 2,2-Diphenyl-1-picrylhydrazyl radical; F-C - Folin–Ciocalteu; GAE - gallic acid equivalent; PBS - Phosphate-buffered saline; PCA - Principal components analysis; QDP - Quantitative Descriptive Profiling; TPC - total polyphenol content; VCEAC - ascorbic acid equivalent

# **Materials and methods**

The scheme of sample preparation is shown in Figure S1.


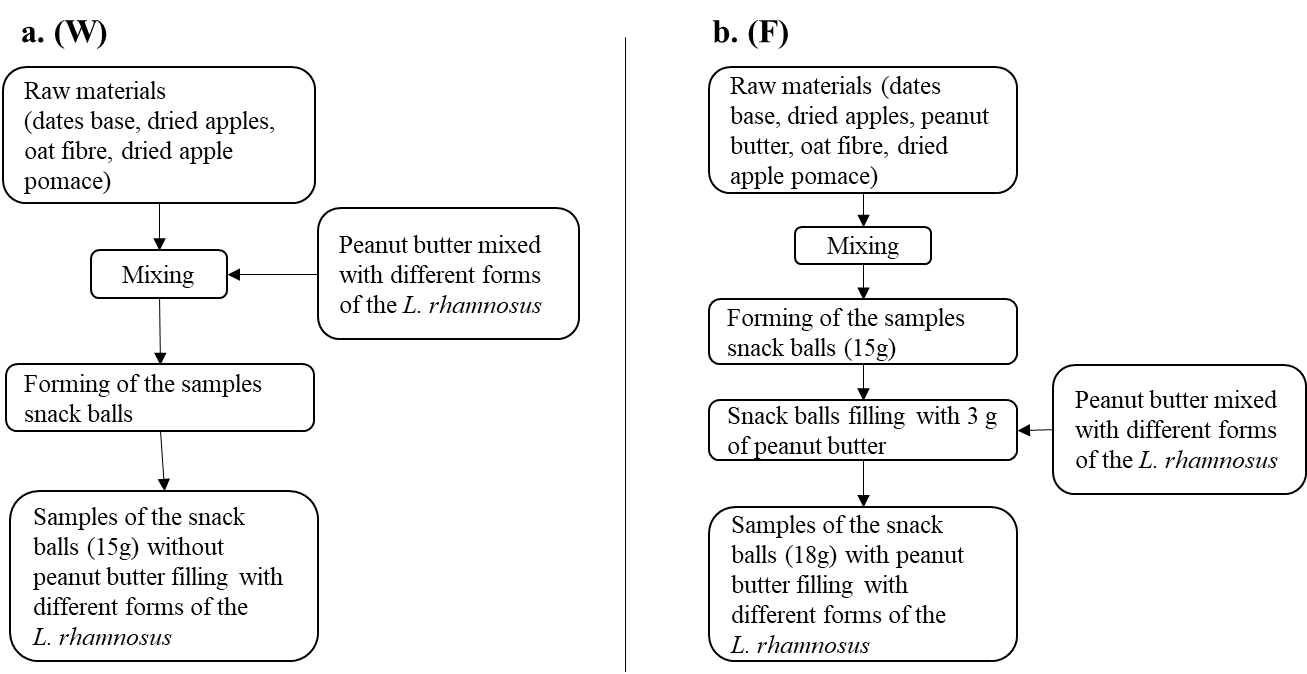


**Fig. S1** Sample preparation scheme; a - samples without peanut butter filling (W); b - samples with a peanut butter filling (F).

- 1. **Probiotic strain**

*Lacticaseibacillus rhamnosus* ATCC 53103 was used in the research. This strain is a well-studied probiotic bacterium known for its high stability and broad range of potential applications [1]. Three different forms of the same strain were applied - biomass, freeze-dried (Dicoflor 6, Bayer, Leverkusen, Germany), and microencapsulated (Floradrop, Aura Herbals, Sopot, Poland). Freeze-dried and microencapsulated bacteria were purchased from a local pharmacy. According to the manufacturer's specifications, the microcapsules were prepared using sodium alginate and cellulose and were spray-dried. The freeze-dried form contains bacteria on a maltodextrin carrier and anti-caking magnesium salts of fatty acids.

To obtain bacterial biomass, *L. rhamnosus* ATCC 53103 was activated from the pure culture (-80℃) and transferred on the MRS agar (Neogen, Heywood, UK) plate by inoculation loop and incubated for 48h anaerobically. Then, a single colony was transferred to 10 mL of MRS broth (Neogen, Heywood, UK) and incubated for 24 h at 37℃. Next, the culture was transferred to 500 mL MRS broth and incubated for 24 h. The bacterial biomass was centrifuged at 10,000 rpm for 10 min. To purify the biomass of MRS residues, the bacterial biomass was washed twice with a sterile 0.9g/100 mL NaCl (Chempur, Piekary Śląskie, Poland) solution and centrifuged at 20℃, 10,000 rpm for 10 min. The obtained bacterial biomass was used for sample preparation immediately after purification.

- 1. **Snack samples preparation**

The study materials were self-made snacks and consisted of 72.9 g/100 g ground dates (KruKam, Wodzisław Śląski, Poland), 11.2 g/100 g ground dried apples (KruKam, Wodzisław Śląski, Poland), 9.3 g/100 g peanut butter (Go Vegan - Jerónimo Martins, Kostrzyn, Poland), 3.6 g/100 g oat fibre (KruKam, Wodzisław Śląski, Poland) and 3.0 g/100 g ground dried apple pomace (Döhler, Kozietuły Nowe, Poland). Before sample preparation, all raw materials were packaged in vacuum bags (Hendi, Robakowo, Polska) and pasteurised in a water bath at 90℃ for 30 min (TSGP20, Thermo Fisher, Massachusetts, USA). All ingredients were weighed and mixed using a Kitchen Aid mixer (5KSM185PS, Whirlpool Corporation, Michigan, USA). Two types of snacks were produced. The first was formed into a ball and weighed 15 g. The second variant differed. It had a 3 g peanut butter filling inside the snack ball and weighed 18 g.

Each form of bacteria (biomass, freeze-dried, or microcapsules) was combined with peanut butter in a separate beaker and mixed thoroughly with a glass rod under sterile conditions. In the case of the variant without peanut butter filling (W), peanut butter with bacteria in various forms was added to the mass and mixed until a homogeneous mass of the samples was obtained. Peanut butter-filled samples (F) were prepared by injecting into the snack ball peanut butter previously inoculated with bacteria, using a sterile syringe (Fig. 1). Control samples were prepared in the same manner without the addition of bacteria. Peanut butter was used as a filling to trap bacteria inside snacks due to its very low water activity and potential bacteria-fixing properties. Peanut butter was also used due to its sensory-creating features.

Bacteria were added at a count of approximately 9.5 log CFU/g. The bacterial count in the snacks after 24 hours of storage must be at least 8 log CFU/g of the sample. Some bacteria were inactivated during snack preparation. These results were based on preliminary tests on the number of bacteria in snacks.

- - 1. **Variants of samples and storage conditions**

Samples were packed in bags made of polyethylene polyamide (Hendi, Robakowo, Polska). Samples were stored for 6 months under refrigeration conditions (4℃) and at ambient temperature (20℃). Table S1 presents variants of the analysed samples.

**Table S1.** Variants of samples used in the study

| Abbreviation name of the sample | Added form of bacteria | Peanut butter filling | Storage temperature (℃) |
| --- | --- | --- | --- |
| C-W (control sample) | none | no addition | no storage, analyzed after production |
| B-W | biomass |  |  |
| F-W | freeze-dried |  |  |
| M-W | microencapsulated |  |  |
| C-W-20 (control sample) | none |  | 20 |
| B-W-20 | biomass |  |  |
| F-W-20 | freeze-dried |  |  |
| M-W-20 | microencapsulated |  |  |
| C-W-4 (control sample) | none |  | 4 |
| B-W-4 | biomass |  |  |
| F-W-4 | freeze-dried |  |  |
| M-W-4 | microencapsulated |  |  |
| C-F (control sample) | none | with addition | no storage, analyzed after production |
| B-F | biomass |  |  |
| F-F | freeze-dried |  |  |
| M-F | microencapsulated |  |  |
| C-F-20 (control sample) | none |  | 20 |
| B-F-20 | biomass |  |  |
| F-F-20 | freeze-dried |  |  |
| M-F-20 | microencapsulated |  |  |
| C-F-4 (control sample) | none |  | 4 |
| B-F-4 | biomass |  |  |
| F-F-4 | freeze-dried |  |  |
| M-F-4 | microencapsulated |  |  |

- 1. **Fibre content**

The content of total dietary fibre, including the soluble and insoluble fibre fractions, was determined using the gravimetric method after enzymatic hydrolysis according to AOAC 991.43, AACC 32-07. Experiments were performed in 3 independent biological replications.

- 1. **Fat content**

The gravimetric method after Soxhlet extraction determined the total fat content according to the PN-A-79011-4:1998 [2]. Experiments were performed in 3 independent biological replications.

- 1. **Water activity**

The water activity was measured by the Hygrolab 3 device (Rotronic AG, Basserdorf, Switzerland). Experiments were performed in 4 independent biological replications.

- 1. **Number of probiotic bacteria during storage**

The probiotic bacteria's survival was tested over a 6-month storage period. Controls without added probiotic bacteria were tested to check for contamination by native lactic acid bacteria. MRS agar (Neogen, Heywood, UK) was used as the growth medium, and samples were diluted in buffered peptone water (Merck, Darmstadt, Germany) before being cultured on the MRS agar. The cultures were incubated under anaerobic conditions generated by anaerogen (Thermo Scientific, MA, USA) for 48 hours at 37°C, and the colonies were counted. The experiments were performed in 3 independent biological replications.

- 1. **Number of the viable count of bacteria, yeast and moulds**

YGC agar (yeast extract glucose chloramphenicol, Neogen, Heywood, UK) was used as a growth medium for yeast and moulds. Nutrient agar (Neogen, Heywood, UK) was used to detect the total viable count of bacteria. Samples were serially diluted in buffered peptone water (Merck, Darmstadt, Germany) and then cultured on the appropriate media. The cultures were incubated aerobically for 24 hours at 37 °C for bacteria and 4 days for yeast and moulds at 25°C. After this time, the growth of microorganisms was assessed. Experiments were performed in 3 independent biological replications.

- 1. **Genetic sequencing**

Pure bacterial cultures were isolated after 6 months of storage from the samples with probiotic addition and from the freeze-dried and microencapsulated bacteria before the experiment to check their purity. DNA isolation was performed by the commercial Genomic Mini AX Bacteria kit (A&A Biotechnology, Gdansk, Poland). To confirm the presence of *Lacticaseibacillus rhamnosus* in the tested samples, PCR amplification of 16S rDNA fragments was performed using primers 27F: 5AGAGTTTGATCMTGGCTCAG-3 and 1492R: 5-GTTACCTTGTTACGACTT-3. The amplification reaction was conducted in an ABI 9700 thermocycler (Thermo Scientific, MA, USA) with the Eurx thermostable OptiTaq polymerase. The PCR reaction conditions were: 1) 95 °C - 3 min; 2) 95°C - 15 s; 3) 54°C - 15 s; 4) 72°C - 90 s; 5) Points 2-4 repeated 35 times; 6) 72°C - 2 min; 7) 10°C - cooling. The PCR product was purified by ExoSap (Thermo Scientific, MA, USA) and sequenced using the kit: BigDye Terminator Mix v3.1 (Thermo Scientific, MA, USA) and ABI3730xl Genetic Analyzer (ABI3730xl Genetic Analyzer) and specific primers. The obtained readings (from bacterial 16S rRNA-specific primers: 341F, 518R and 928F) were assembled into contigs to receive a consensus sequence. Using the BLAST program, the obtained sequence consensus was compared with the NCBI-GeneBank database. The experiment was performed in a single biological replicate.

- 1. **The potential synbiotic properties**

To analyse whether the designed samples stimulate the growth of L. rhamnosus ATCC 53103, bacterial cultures were grown based on water extracts of samples. Total fibre content, non-dissolving fibre, and soluble fibre were analysed. A mass of snacks with changed proportions of ingredients was used in a few combinations. Different combinations of ingredient proportions were tested, but peanut butter was excluded due to difficulties encountered during sample preparation, where a fatty layer precipitated. The components of the samples were mixed with a Kitchen Aid mixer, formed snack balls (15 g) and extracted. The sample compositions used for this study stage are presented in Table S2. Extraction was performed according to Wang *et al*. [3] with modification. Ten grams of each sample was homogenized in 90 mL of deionized water. Then the samples were extracted in a water bath for 6 h at a temperature of 65℃ with continuous stirring of 250 rpm. Next, the samples were sterilized at 121℃ for 15 min. Water extracts prepared this way were used as a growth medium for bacteria. One hundred µL of 24 h culture of the *L. rhamnosus* ATCC 53103 was added to 10 mL of the sample's extracts. The preparation of the culture was described in the 2.1 section. The number of bacteria was determined at 0, 6, 12, 24, 36, 48, and 60 h of cultivation by plate counting method described in the 2.6 section. Experiments were performed in 3 independent biological replications.

**Table S2.** Variants of samples related to potential synbiotic properties

| Sample code | Ingredients g/100 g | | | |
| --- | --- | --- | --- | --- |
|  | Ground dates | Ground dried apples | Oat fibre | Ground-dried apple pomace |
| P1 | 87.0 | 13.0 | - | - |
| P2 | 83.6 | 13.0 | 3.6 | - |
| P3 | 84.0 | 13.0 | - | 3.0 |
| P4 | 80.4 | 13.0 | 3.6 | 3.0 |

- 1. **Total polyphenol content and antioxidant properties**

The samples were ground to a homogeneous mass using a laboratory grinder. One gram of each sample was mixed in 50 mL of 80% (v/v) methanol (Chempur, Piekary Śląskie, Poland). The samples were then extracted in an ultrasonic bath at 30 °C for 15 min. The samples were centrifuged using an Eppendorf 5804 R centrifuge (10 min, 10000 rpm, 0 °C). The extracts were stored at -20 °C in the dark until use. Analysis of TPC, ABTS and DPPH was performed directly after snack preparation and storage for 6 months according to Lou *et al.* [19].

- - 1. **Total polyphenol content**

The extracts of the samples were diluted in demineralised water. 20 µL of the prepared sample dilution was poured into the polystyrene 96-well plate (NEST Biotechnology, Wuxi, China) and 100 µL of F-C reagent (Chempur, Piekary Śląskie, Poland) was added. The plate was left for 5 min at room temperature in a dark place. 80 μL of the 7.5% sodium carbonate (Chempur, Piekary Śląskie, Poland) solution in demineralised water was poured into the wells, mixed at 150 rpm for 5 min and left for 2 hours in a dark place. The samples were measured at wavelength λ = 750 nm using the SpectraMax iD3 reader (Molecular Devices, CA, USA). Results are expressed as gallic acid equivalent (GAE). Absorbance values were converted using the standard curve (y=0.0193+0.3766x; R^2^=0.999). Experiments were performed in 4 independent biological replications.

- - 1. **ABTS**

The antioxidant activity was measured using the radicals ABTS (Sigma-Aldrich, Poznań, Poland). ABTS was prepared 24 h before the determination by mixing powder ABTS radicals (7 mM/L) with potassium persulfate salt (2.45 mM/L) (Sigma-Aldrich, Poznań, Poland) and stored for 24h at 20℃. The ABTS solution was diluted with PBS (Sigma-Aldrich, Poznań, Poland) the absorbance of 0.7 ± 0.02 at λ = 734 nm. Fifty μL of each of the test sample solutions and 150 μL of the ABTS radical solution were poured into a well of a 96-well polystyrene plate. The reaction was run for 6 minutes and then measured at 734 nm with a SpectraMax iD3 reader. Results are expressed as ascorbic acid equivalent (VCEAC). Absorbance values were converted using the standard curve (y= 0.7157-391.1242x; R^2^=0.996). Experiments were performed in 4 independent biological replications.

- - 1. **DPPH**

The antioxidant activity was determined using the synthetic radical DPPH (Sigma-Aldrich, Poznań, Poland). The DPPH solution was stored in the dark. The obtained solution was diluted so that its absorbance at the wavelength λ = 517 nm was 1.1 ± 0.05 using methanol as a solvent. The initial absorbance of the DPPH radical solution was measured by adding 150 µL of the DPPH solution and 5 µL of methanol. The test sample contained 150 µL of DPPH solution and 5 µL of the sample extract, the absorbance was measured after 30 min from the initiation of the reaction. The sample was stored in the dark with constant shaking at 150 rpm during incubation. Absorbance was measured at a wavelength of λ = 517 nm. Results are expressed as ascorbic acid equivalent (VCEAC). Absorbance values were converted using the standard curve (y=1.1346-249.6462x; R^2^=0.997). Experiments were performed in 4 independent biological replications.

- 1. **Texture analysis**

The texture tests were based on cutting force and deformation tests at maximum cutting force using a Warner-Braztler attachment equipped with a flat knife. The tests were performed using the Zwick1120 testing machine (Zwick, Germany). The parameters of the analysis were: the distance of the knife to the table equal to 30 mm; the speed of setting the initial position of the knife was 400 mm/min; the initial force (marking the beginning of the test) was 0.5 N; pre-force setting speed was 200 mm/min; the test speed was 50 mm/min; the end of the test was 50% of Fmax or deformation of 25 mm. Only control samples were used for this experiment. Experiments were performed in 3 independent biological replicates.

- 1. **Sensory analysis**
     1. **Quantitative Descriptive Profiling and Trained Panel Evaluation**

The tests were conducted on samples within 24 hours of production and after 3 and 6 months of storage, using only control samples for this experiment. The sensory profile of the samples was established by trained panellists using the QDP method [5]. These panellists possessed extensive theoretical and practical experience in sensory procedures and sensory evaluation of various food products.

QDP analysis descriptors were defined during a panel discussion and verified in a preliminary session. Finally, 16 sensory attributes were measured. These are shown in Figure 5 (main manuscript). The panellists determined the overall sensory quality based on all the characteristics (low - very high). An unstructured linear scale of 100 mm was used, which was converted into numerical values (0-10 conventional units c.u.). The anchor values of the attributes tested were as follows: none - very strong. There were two exceptions for colour (dark–bright) and texture (low-high). Two independent sessions (replications) were performed. The average result of the method was based on 16 individual results. The samples were coded separately for each evaluation with three-digit codes. The samples were served in random order and analysed at different times to avoid the influence of an earlier sample on a later one. Between the subsequent evaluations, the assessors received water to neutralize the taste.

- - 1. **Consumers’ sensory test and consumer panel**

A 9-point hedonic scale was used for all attributes analysed according to Meilgaard *et al.* [6]. The attributes evaluated were appearance, consistency, taste and overall liking. All participants rated samples from 1 (extremely dislike) to 9 (extremely like). Only control samples were used in this analysis. The test was conducted on samples within 1 day of production. The samples were randomly coded and the consumer was given all the samples independently in a closed transparent container. Consumers' liking of the sample snack balls was assessed by 40 participants who reported eating sweet snacks and had no dietary restrictions. Participants were between 18 and 24 years old and represented mainly young adult consumers, residents of cities with more than 500,000 inhabitants, from the central part of Poland. All participants gave their voluntary consent to participate in the study in the form of a written agreement.

- 1. **Statistical analysis**

Statistical analysis was conducted using the Statistica 13.3 program (StatSoft, Krakow, Polska). Descriptive analysis was performed (mean, standard deviation). The homogeneity of variance and the normality of the distribution of results were checked. Variance analysis was performed with Tukey's post hoc test. In cases where ANOVA could not be used, a Student's t-test was performed. PCA was performed based on the covariance matrix.

**References for methods**

1. Capurso L (2019) Thirty Years of Lactobacillus rhamnosus GG: A Review. J Clin Gastroenterol 53 Suppl 1:S1–S41. https://doi.org/10.1097/MCG.0000000000001170

2. Polish Standard. PN-A-79011-4:1998. Polish Committee for Standardization. Dry food mixes – Test methods – Determination of fat content. https://sklep.pkn.pl/pn-a-79011-4-1998p.html. Accessed 24 Feb 2024

3. Wang S, Fang Y, Xu Y, *et al* (2022) The effects of different extraction methods on physicochemical, functional and physiological properties of soluble and insoluble dietary fiber from Rubus chingiiHu. fruits. J Funct Food 93:105081. https://doi.org/10.1016/j.jff.2022.105081

4. Lou H, Hu Y, Zhang L, *et al* (2012) Nondestructive evaluation of the changes of total flavonoid, total phenols, ABTS and DPPH radical scavenging activities, and sugars during mulberry (*Morus alba L.*) fruits development by chlorophyll fluorescence and RGB intensity values. LWT 47:19–24. https://doi.org/10.1016/j.lwt.2012.01.008

5. ISO 13299:2016 (2016) Sensory Analysis-Methodology-General Guidance for Establishing a Sensory Profile; ISO: Geneva, Switzerland.

6. Meilgaard MC, Carr BT, Carr BT (2007) Affective Tests: Consumer Tests and In-House Panel Acceptance Tests. In: Sensory Evaluation Techniques, 4th ed. CRC Press

# **Results (DNA sequencing, moulds, yeast and total viable count of bacteria)**

**Table S3.** The results of coverage of the obtained sequence of the 16S rDNA gene fragment in the tested samples; (n=1)

| Sample | Sequence coverage (%) | Identified species of bacteria |
| --- | --- | --- |
| Biomass | 100 | *L. rhamnosus* |
| Freeze-dried | 100 | *L. rhamnosus* |
| Microcapsules | 100 | *L. rhamnosus* |
| B-W-4 | 100 | *L. rhamnosus* |
| B-F-4 | 100 | *L. rhamnosus* |
| F-W-4 | 100 | *L. rhamnosus* |
| F-F-4 | 100 | *L. rhamnosus* |
| M-W-4 | 100 | *L. rhamnosus* |
| M-F-4 | 100 | *L. rhamnosus* |
| B-F-20 | 100 | *L. rhamnosus* |
| F-F-20 | 100 | *L. rhamnosus* |
| M-F-20 | 100 | *L. rhamnosus* |

Explanations: biomass, freeze-dried and, microcapsules mean the sources of the isolation of the selective bacterial colony; sources of bacteria are described in the material and methods section

**Table S4.** Presence of total viable count of bacteria, yeasts and moulds in tested samples during storage; (n=3)

| Sample | Total viable count of bacteria (CFU/g) | | | | | | | Yeast and moulds (CFU/g) | | | | | | |
| --- | --- | --- | --- | --- | --- | --- | --- | --- | --- | --- | --- | --- | --- | --- |
|  | Months of storage | | | | | | | | | | | | | |
|  | 0 | 1 | 2 | 3 | 4 | 5 | 6 | 0 | 1 | 2 | 3 | 4 | 5 | 6 |
| C-W | <10 | | | | | | | <10 | | | | | | |
| B-W | <10 | | | | | | | <10 | | | | | | |
| F-W | <10 | | | | | | | <10 | | | | | | |
| M-W | <10 | | | | | | | <10 | | | | | | |
| C-W-20 | <10 | | | | | | | <10 | | | | | | |
| B-W-20 | <10 | | | | | | | <10 | | | | | | |
| F-W-20 | <10 | | | | | | | <10 | | | | | | |
| M-W-20 | <10 | | | | | | | <10 | | | | | | |
| C-W-4 | <10 | | | | | | | <10 | | | | | | |
| B-W-4 | <10 | | | | | | | <10 | | | | | | |
| F-W-4 | <10 | | | | | | | <10 | | | | | | |
| M-W-4 | <10 | | | | | | | <10 | | | | | | |
| C-F | <10 | | | | | | | <10 | | | | | | |
| B-F | <10 | | | | | | | <10 | | | | | | |
| F-F | <10 | | | | | | | <10 | | | | | | |
| M-F | <10 | | | | | | | <10 | | | | | | |
| C-F-20 | <10 | | | | | | | <10 | | | | | | |
| B-F-20 | <10 | | | | | | | <10 | | | | | | |
| F-F-20 | <10 | | | | | | | <10 | | | | | | |
| M-F-20 | <10 | | | | | | | <10 | | | | | | |
| C-F-4 | <10 | | | | | | | <10 | | | | | | |
| B-F-4 | <10 | | | | | | | <10 | | | | | | |
| F-F-4 | <10 | | | | | | | <10 | | | | | | |
| M-F-4 | <10 | | | | | | | <10 | | | | | | |
